# SARS-CoV-2 Assembly: Gaining Infectivity and Beyond

**DOI:** 10.3390/v16111648

**Published:** 2024-10-22

**Authors:** Harshita Katiyar, Ariana Arduini, Yichen Li, Chen Liang

**Affiliations:** 1 Lady Davis Institute, Jewish General Hospital, Montreal, QC H3T 1E2, Canada; harshita.katiyar@mail.mcgill.ca (H.K.); ariana.arduini@mail.mcgill.ca (A.A.); yichen.li2@mail.mcgill.ca (Y.L.); 2 Department of Microbiology and Immunology, McGill University, Montreal, QC H3A 2B4, Canada; 3 Department of Medicine, McGill University, Montreal, QC H3G 2M1, Canada

**Keywords:** SARS-CoV-2, viral assembly, structural proteins, virus–host interactions

## Abstract

Severe acute respiratory syndrome coronavirus 2 (SARS-CoV-2) was responsible for causing the COVID-19 pandemic. Intensive research has illuminated the complex biology of SARS-CoV-2 and its continuous evolution during and after the COVID-19 pandemic. While much attention has been paid to the structure and functions of the viral spike protein and the entry step of viral infection, partly because these are targets for neutralizing antibodies and COVID-19 vaccines, the later stages of SARS-CoV-2 replication, including the assembly and egress of viral progenies, remain poorly characterized. This includes insight into how the activities of the viral structural proteins are orchestrated spatially and temporally, which cellular proteins are assimilated by the virus to assist viral assembly, and how SARS-CoV-2 counters and evades the cellular mechanisms antagonizing virus assembly. In addition to becoming infectious, SARS-CoV-2 progenies also need to survive the hostile innate and adaptive immune mechanisms, such as recognition by neutralizing antibodies. This review offers an updated summary of the roles of SARS-CoV-2 structural proteins in viral assembly, the regulation of assembly by viral and cellular factors, and the cellular mechanisms that restrict this process. Knowledge of these key events often reveals the vulnerabilities of SARS-CoV-2 and aids in the development of effective antiviral therapeutics.

## 1. Introduction

The emergence of severe acute respiratory syndrome coronavirus 2 (SARS-CoV-2), the causative agent of the global COVID-19 pandemic, has profoundly impacted societies, economies, and healthcare systems worldwide [1,2]. Since its initial outbreak in Wuhan, China, in December 2019, this virus has rapidly disseminated, infecting more than 776 million individuals and causing over 7 million fatalities worldwide [3]. SARS-CoV-2 is a member of the Coronaviridae family that also includes the highly pathogenic severe acute respiratory syndrome coronavirus (SARS-CoV) and Middle East respiratory syndrome coronavirus (MERS-CoV), both of which have caused notable outbreaks in the past two decades [2,4,5]. The clinical presentations of SARS-CoV-2 infection can range from mild flu-like symptoms to respiratory distress syndrome, multi-organ failure, and death in severe cases [6,7,8]. Despite the success of COVID-19 vaccines and antiviral drugs in preventing and treating severe disease, SARS-CoV-2 continues to circulate in the population due to the constant emergence of highly transmissible and immune evasive variants [9,10].

While the early stages of SARS-CoV-2 infection, including viral entry, have been extensively studied for their relevance as targets of approved vaccines, the later stages of virion assembly remain relatively less examined [11]. In line with this, the currently approved drugs target these early stages, such as Paxlovid (nirmatrelvir coupled with ritonavir), a viral protease inhibitor that blocks viral non-structural polyprotein cleavage, or Molnupiravir and Remdesivir, which inhibit SARS-CoV-2 RNA-dependent RNA polymerase (RdRp) and disrupt RNA synthesis [12,13,14]. However, SARS-CoV-2 assembly, which generates infectious virus particles, is the final and critical stage of its replication [15]. Viral structural proteins are essential in this process for assembling SARS-CoV-2 and include the following: the spike (S), protruding from the virus surface and mediating viral entry into host cells; the envelope (E) and membrane (M) proteins, both embedded within the viral membrane; and the nucleocapsid (N), encapsulating the single-stranded positive-sense viral RNA genome into new virions [15,16,17].

This review is aimed at providing an updated overview of the known functions of these viral structural proteins in the assembly of SARS-CoV-2 particles, while also addressing the contributions of other viral proteins, the temporal and spatial regulation of viral assembly, and the cellular restriction mechanisms that target this crucial step in viral replication. Understanding the molecular details of SARS-CoV-2 assembly is essential for identifying potential drug targets that disrupt key steps in the assembly process, ultimately inhibiting the virus’s ability to replicate and spread.

## 2. Assembly and the Final Stages of SARS-CoV-2 Replication

**Figure 1:**
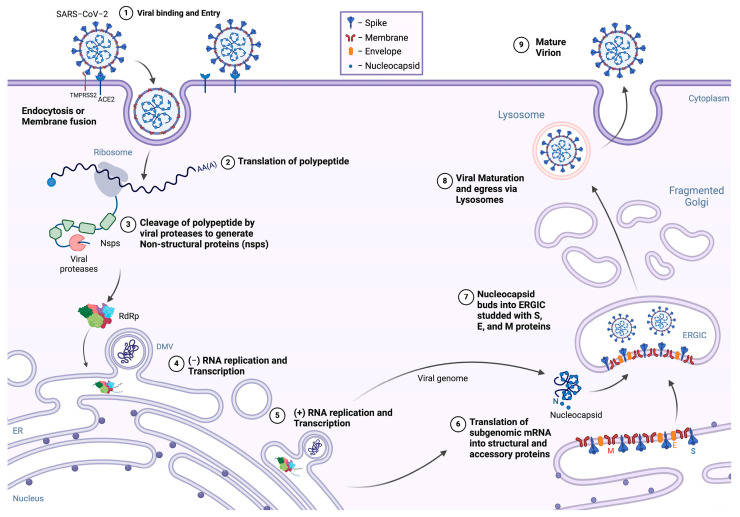
Schematic of the SARS-CoV-2 replication cycle. (1) SARS-CoV-2 initiates infection by binding to ACE2 on the host cell surface and enters either via membrane fusion at the plasma membrane in the presence of TMPRSS2 or via endocytosis using cathepsins. (2) The viral positive-sense RNA genome is released into the cytoplasm and translated by host ribosomes to produce polypeptides. (3) These are subsequently cleaved by viral proteases to form the NSPs which form the RdRp complex and double-membrane vesicles (DMVs). (4) Negative-sense RNAs are synthesized using the positive-sense RNA genome as a template. (5) These negative-sense RNAs serve as templates for synthesizing new positive-sense genomic and subgenomic RNAs. (6) The subgenomic RNAs are translated into structural and accessory proteins. (7) The genomic RNA is bound by the nucleocapsid protein, while structural proteins S, E, and M are embedded on the endoplasmic reticulum–Golgi intermediate compartment (ERGIC). (8–9) Virions assemble in the ERGIC and are transported out of the cell via exocytosis. Created in BioRender.com, accessed on 21 October 2024.

The production of SARS-CoV-2 progeny marks the end of one replication cycle [11] (Figure 1). As the final stage of infection, assembly depends on earlier steps that not only synthesize balanced amounts of viral structural proteins and genomic RNA, but also alter the cellular environment to promote the assembly and release of viral progeny.

SARS-CoV-2 infection begins with the binding of the viral S protein to the host cell receptor angiotensin-converting enzyme 2 (ACE2) [1,18,19]. Interaction with ACE2 exposes a site in S, called S2’, which is cleaved by cellular proteases such as TMPRSS2 or lysosomal cathepsins B and L [20,21]. Depending on the availability of these proteases, SARS-CoV-2 can complete membrane fusion, and thus entry, at the plasma membrane (TMPRSS2-dependent) or through endocytosis in lysosomes (cathepsin-dependent) [18,22]. Several other cell surface proteins, such as Asialoglycoprotein receptor-1 (ASGR1), Kringle Containing Transmembrane Protein 1 (KREMEN1), Scavenger receptor B type 1 (SR-B1), Neuropilin-1 (NRP-1), Transmembrane protein 106B (TMEM106B), the transferrin receptor (TFR), and Niemann-Pick C1 (NPC1), have also been implicated in S interactions at the cell surface, and hence, in SARS-CoV-2 entry [23,24,25,26,27,28,29,30,31]. Their exact roles in relation to ACE2 and in SARS-CoV-2 pathogenesis and transmission await further investigation.

After delivery into the cytoplasm, SARS-CoV-2 genomic RNA (gRNA) can readily engage ribosomes to synthesize viral non-structural proteins (NSPs). These NSPs carry out essential functions. For instance, viral proteases NSP3 and NSP5 cleave the newly synthesized viral polyprotein to generate mature and functional proteins. Moreover, NSP3, NSP4, and NSP6 associate with the endoplasmic reticulum (ER) to drive the formation of double-membrane vesicles (DMVs). DMVs are the primary site for coronaviral RNA synthesis, whereas alternative replication organelles, like convoluted membranes and single-membrane vesicles, have also been observed [32,33,34]. This compartmentalization potentially helps SARS-CoV-2 to evade innate immune detection by shielding the newly synthesized dsRNA from sensing by host pattern recognition receptors like melanoma differentiation-associated protein 5 (MDA5) [34,35]. RNA synthesis occurs discontinuously in these DMVs, with the transcription complex synthesizing both full-length viral gRNA and shorter subgenomic RNA (sgRNA) [4,36,37]. Additionally, the viral RNA-dependent RNA polymerase (RdRp) NSP12, together with NSP7-NSP10, NSP13, NSP14, and NSP16, transcribes and replicates viral RNA in DMVs [38,39,40,41,42,43]. The newly synthesized SARS-CoV-2 gRNA and sgRNA are then transported out of DMVs for subsequent translation and virion assembly, a process recently found to be mediated by a molecular pore on the DMVs formed primarily by NSP3 and NSP4 [37,44]. SARS-CoV-2, like other coronaviruses, requires mechanisms to reach a balance between the full-length gRNA and sgRNA, both of which are critical for viral assembly. Nevertheless, these mechanisms are not fully defined.

During later stages of assembly, SARS-CoV-2 structural proteins (M, E, N, and S), as well as accessory proteins, are synthesized from viral sgRNAs [4]. The relative abundance of these sgRNAs, and consequently the structural proteins, remains unclear, but may affect the stoichiometry of these components in the virion, ultimately affecting viral infectivity. It is also not well understood how the extensive rearrangement of the ER during DMV formation affects the downstream synthesis of viral M, E, S, and other membrane-bound viral and cellular proteins, nor the involvement of the Golgi in virus assembly. The M, E, and S proteins are synthesized in the ER and trafficked to the ER–Golgi intermediate compartment (ERGIC), where they assemble with the viral N protein and genomic RNA to form new virions. These newly formed SARS-CoV-2 particles are then reported to egress via the lysosomal secretory pathway [45]. The detailed mechanisms are discussed in the following sections.

## 3. Experimental Systems to Study SARS-CoV-2 Assembly

The development of various experimental systems has been crucial to our understanding of SARS-CoV-2 assembly by enabling the detailed study of the molecular mechanisms and pathways involved in viral particle formation. In SARS-CoV-2-infected cells, the trafficking and localization of viral structural proteins and gRNA can be characterized by immunofluorescence and imaging techniques, while the formation of progeny viruses can be visualized by electron microscopy [33,46,47]. These methods have revealed key mechanisms of the assembly process, including the assembly site and egress pathway of SARS-CoV-2 particles, and membrane compartments where these virus particles are contained.

On the other hand, the functional investigation of viral structural proteins and their interactions with cellular factors can be challenging, and this is partly due to the complexities of performing reverse genetics to introduce mutations into recombinant viruses, as well as because of the overlapping roles of several cellular proteins throughout the viral replication cycle. These difficulties can therefore be circumvented with the virus-like particle (VLP) system, which can be safely handled in biosafety level 2 facilities. VLPs, produced through the co-expression of the four structural proteins M, E, N, and S, morphologically resemble live SARS-CoV-2 viral particles [48] and can induce protective immunity as vaccines [49,50]. Notably, an important improvement of the VLP assay includes the incorporation of a firefly luciferase reporter mRNA containing the SARS-CoV-2 RNA packaging signals, thus producing luciferase upon delivery into ACE2-positive target cells and providing a functional luminescence readout [51].

In addition to live SARS-CoV-2 virus and VLP systems, cell-free biochemical assays using purified viral proteins and lipids also offer mechanistic insights into the assembly processes [52,53,54]. Techniques such as cryo-electron microscopy (cryo-EM) and X-ray crystallography have provided atomic-level details of structural protein interactions essential for virus assembly [36,46,55,56]. Computational modeling and in silico studies complement the experimental approaches discussed above by predicting protein–protein interactions and membrane dynamics during assembly [57,58]. Collectively, these experimental approaches have allowed for the elucidation of what is known about the molecular mechanisms and pathways driving SARS-CoV-2 assembly.

## 4. Building Blocks of SARS-CoV-2 Particles

**Figure 2:**
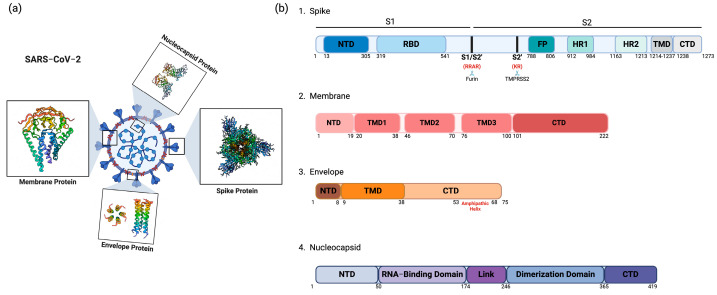
SARS-CoV-2 structural proteins. (**a**) Schematic of the SARS-CoV-2 particle and its structural proteins, depicting its spherical, enveloped form with protruding spike (S) proteins (PDB: 6VXX). The envelope (E) (PDB: 7K3G) and membrane (M) (PDB: 8CTK) proteins are embedded within the lipid bilayer, providing structural integrity and shape to the virus. Inside the virion, the nucleocapsid (N) protein (PDB: 8FG2) encapsulates the positive-sense RNA genome, forming the ribonucleoprotein (RNP) complex. (**b**) Domain organization of the four structural proteins, with amino acid positions denoted. Key functional sites are depicted, including the Furin and TMPRSS2 cleavage sites in spike, the NTD (N-terminal domain), RBD (receptor-binding domain), FP (fusion peptide), HR (heptad repeat), TMD (transmembrane domain), and CTD (C-terminal domain). Created in BioRender.com, accessed on 21 October 2024.

The viral E, M, N, and S proteins, along with the viral genomic RNA, are the fundamental components comprising an infectious SARS-CoV-2 particle (Figure 2a). In addition to these, a significant amount of accessory factor ORF3a, a homolog of the M protein, has also been found in SARS-CoV-2 particles, and emerging research has begun to elucidate cellular proteins that either support or hinder SARS-CoV-2 particle assembly [59].

Firstly, the M protein drives the assembly of SARS-CoV-2 particles [4]. It serves two main functions in this context: self-multimerization to induce membrane curvature for the formation of the budding particle, and the interaction and recruitment of E, S, N, and other viral and cellular proteins into the progeny particle [60]. As such, the M protein also dictates the intracellular site of SARS-CoV-2 particle assembly. The M protein is highly conserved across betacoronaviruses and within SARS-CoV-2 variants [61]. Structurally, it exhibits a triple-helix bundle shape with three transmembrane (TM) domains, a glycosylated N-terminal domain (NTD) in the lumen, and an extended C-terminal tail in the cytoplasm (Figure 2b) [62]. Its carboxy-terminal tail interacts with the N-RNA complex, which in turn stabilizes the M tail and maintains an optimal conformation for particle assembly [62]. Important trafficking motifs have been identified in the M carboxy-terminal tail of SARS-CoV and MERS-CoV, including the negatively charged C-terminal (SWWSFNPETNNL) domain, the trans-Golgi network (TGN) retention motif (KxGxYR), and the ER exit motif (DxE), altogether regulating the intracellular trafficking of M to the assembly site [63,64]. Additionally, M transmembrane domain interactions promote S protein incorporation into forming particles via M-S interactions [65,66,67]. Notably, multiple studies show that SARS-CoV-2 VLP formation mandates the co-expression of the M protein alongside either the E or N protein [48,49]. Altogether, this suggests that the oligomerization of M and the subsequent induction of membrane curvature may depend on interactions with another viral or host protein to initiate the assembly process.

The E protein also promotes virus assembly through its interactions with the M protein, with its viroporin activity, and with its ability to induce membrane curvature, altogether making it a key structural component in the final stages of this process [65]. E is a small transmembrane protein composed of 75 amino acids, with an N-terminal region of 12 amino acids, a hydrophobic TM domain of 25 amino acids, and a lengthy hydrophilic C-terminal domain (CTD) (Figure 2b) [68]. The TM domain can pentamerize to create an ion-channel pore, and the viroporin activity is involved in the deacidification of intracellular compartments, aiding efficient viral egress [69,70,71,72,73,74,75,76]. A previous study observed that the E protein can directly induce membrane curvature via the amphiphilic nature of its CTD [77]. This property was also found to be conserved across E proteins of related betacoronaviruses [61]. Hence, the E protein could play an important role in viral budding and in membrane scission to complete the formation of virus particles, in analogy to the role of the M2 protein of influenza viruses [78,79].

Also central to viral assembly, the N protein packages viral gRNA into newly formed particles. To accomplish this, N selectively binds viral gRNA while excluding viral sgRNA and cellular RNA, a complex termed the ribonucleoprotein (RNP), and then interacts with the M protein to ensure the proper packaging of this viral RNP into the virus particle [52]. The N protein is a 46 kDa RNA-binding protein with 419 amino acids divided into five domains: the NTD, an RNA-binding domain (RBD), a central linker (LINK), a dimerization domain, and the CTD, all well conserved across coronaviruses (Figure 2b) [80,81]. Important to its function, the N protein RBD binds the viral gRNA with high affinity, and leads to the formation of N-RNA complexes which align along the 30 kb viral RNA to create a “beads-on-a-string” structure [82]. Moreover, the central domain and RNA binding regions of N together promote phase separation with viral RNA, allowing for the transition of N-RNA complexes from a liquid-like to gel-like state during packaging to form biomolecular condensates (BMC) which help concentrate and form RNPs [52,80,82,83,84]. RNP formation is an important step, and it has recently been targeted for its therapeutic potential using a small molecule called PAV-104, which was found to inhibit assembly by preventing N oligomerization. However, further clinical data are required to assess the efficacy of this drug [85].

While not essential for the assembly process itself, the S protein is required for SARS-CoV-2 infectivity as it engages the ACE2 receptor and completes virus entry [86]. The S protein forms a trimeric crown-like structure, visible under electron microscopy and leading to the name “coronavirus” [87]. It is composed of three identical monomers, each about 180–200 kDa in size, and is 1273 aa long [88,89]. The SARS-CoV-2 spike protein consists of two functional subunits: S1 (14–685 aa) for receptor binding, and S2 (686–1273 aa) for membrane fusion (Figure 2b) [89]. These subunits are linked by a Furin cleavage site (RRAR), which primes the S protein and facilitates its interaction with ACE2. Shortly after entering the human population, the S protein acquired the D614G mutation, which stabilizes the Furin-cleaved S1/S2 heterodimer and increases S incorporation into virions [90]. Although absent in the SARS-CoV S protein, the Furin cleavage site is not unique to SARS-CoV-2 S and is also present in other betacoronaviruses, such as MERS-CoV and common cold coronaviruses HCoV-HKU-1 and HCoV-OC43 [91]. The S protein also possesses an ER-retention signal at its C-terminus, which prevents its cell-surface trafficking and immune detection, thus enriching S at assembly sites for incorporation into viral particles [22,92,93]. Moreover, the S protein is heavily glycosylated, containing 23 N-linked glycosylation sites [94,95]. The S protein’s acquisition of the appropriate glycosylation profile is essential for its ability to recognize and bind to host cell receptors during viral entry, for immune evasion, and for its other properties which will be discussed in later sections [95,96].

Finally, viral gRNA is the cargo of SARS-CoV-2 particles, which is delivered into target cells to produce more viral progeny [82]. To ensure its selective packaging over sgRNAs and cellular RNAs, the viral gRNA has sequences that are absent in other RNA molecules and that bind with high affinity to the viral N protein. One study found that these sequences include the 5′ UTR and a fragment close to the 3′ terminus of ORF1ab [51]. Similar viral RNA packaging signals have also been described for other coronaviruses, such as the PS580 RNA sequence located close to the 3′ terminus of ORF1b (nt 19,715 to 20,294) in SARS-CoV [97]. The formation of the RNP in the cytoplasm not only shields the viral RNA from cellular defenses but also enables the trafficking of viral RNA to assembly sites, where it interacts with structural proteins for incorporation into the developing virions.

## 5. The Saga of SARS-CoV-2 Assembly

**Figure 3:**
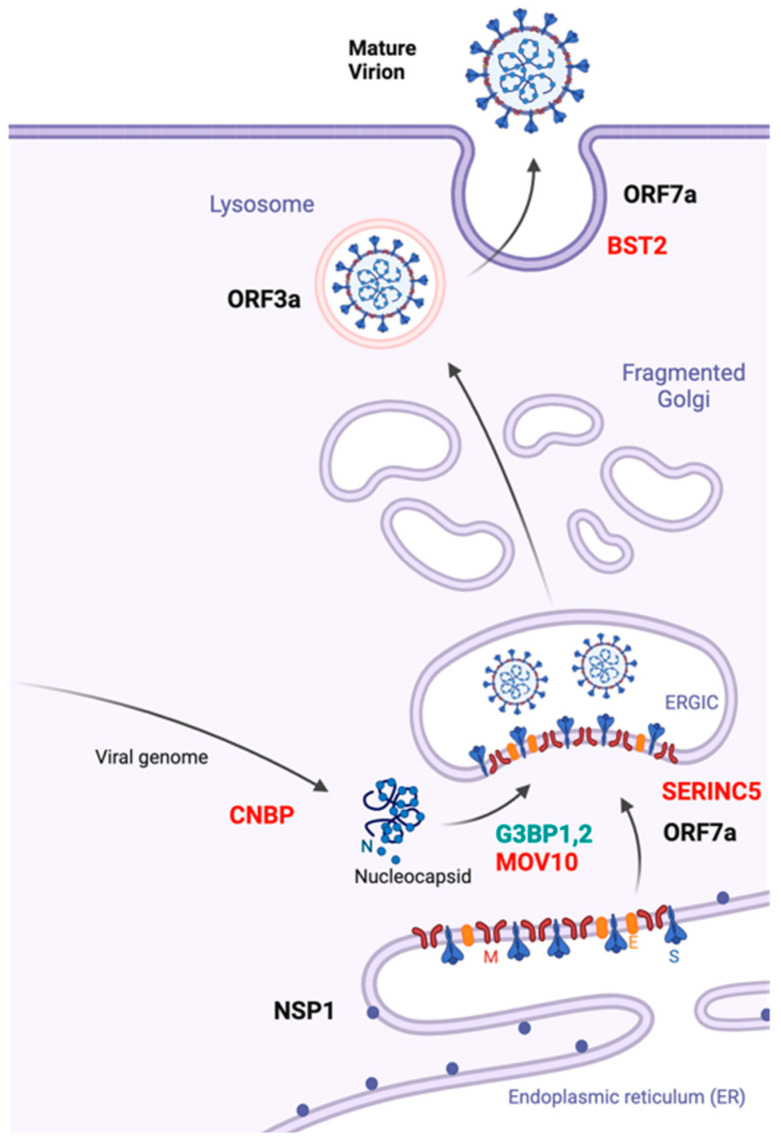
SARS-CoV-2 assembly and its regulation. Viral structural proteins S, E, and M are synthesized and processed in the endoplasmic reticulum (ER). The genomic RNA, bound by the N protein, joins the S, E, and M proteins at the ER–Golgi intermediate compartment (ERGIC) to form new virions. Shown are host factors that have been reported to restrict (in red) or promote (in green) SARS-CoV-2 assembly. Viral accessory protein ORF7a antagonization of SERINC5 and BST-2 is depicted. Created in BioRender.com, accessed on 26 September 2024.

The formation of infectious SARS-CoV-2 particles follows a coordinated sequence of events that occur within specific membrane compartments, such as the ERGIC. These events include the trafficking of viral structural proteins and viral gRNA to sites of assembly, virus particle formation, membrane scission, and finally, egress of progeny virions from the infected cells (Figure 3). Each step of this directional process is either facilitated or antagonized by host cellular machineries. Although recent advancements have shed some light on the details of this complex process, many outstanding questions remain.

### 5.1. The Grand Gathering

Multiple studies have indicated that the minimal components M, E, S, and N proteins are sufficient to form VLPs, and hence, SARS-CoV-2 particles. Therefore, when these structural proteins are expressed from separate plasmids, transfected cells can produce and release VLPs into the culture media, although viral accessory proteins may also assist in achieving optimal infectivity [48,49,65]. Once these essential viral components are synthesized, they need to find one another to form virions. They likely act in two groups based on where they are synthesized within cells.

The first group comprises the M, E, and S proteins and is synthesized in the ER, while the second group includes the N protein and viral gRNA in the cytosol. Given that the sgRNAs encoding M, E, and S are transcribed in DMVs and share the same 5′ and 3′ UTRs, which bind proteins involved in mRNA trafficking and translation, it is likely that these mRNAs exit the DMVs together and hence coordinate protein synthesis at the same or proximal ER sites. One apparent advantage of their spatial proximity is that it facilitates interactions between the M, E, and S, and that they may be sorted into the same COP-II vesicles, responsible for protein transport from the ER to the Golgi. While the M, E, and S proteins interact via their transmembrane domains and intracellular sequences, the molecular details remain to be resolved [16,57]. Currently, it is believed that each of these viral membrane proteins bear short peptide sequences in their cytoplasmic regions to regulate their trafficking between cellular membrane compartments. For example, the S protein possesses an ER-retention signal (“KxxHxx”) which interacts with trafficking components to retrieve the S protein from the ERGIC and cis-Golgi back to the ER [98,99]. This retrieval activity is suppressed when either the M or E proteins are present, since their interactions allow S to be sequestered in the Golgi [65,99].

The N protein, on the other hand, is synthesized by ribosomes in the cytosol. In SARS-CoV-2-infected cells, electron microscopy has revealed that the N protein localizes to the cytoplasmic face of the pore in the viral DMVs and associates with viral RNA exiting these sites [36,46]. This RNP must then find the M/S/E clusters in order to get packaged into the newly assembled particles. N binds to the cytoplasmic tail of M [52], which not only promotes the packaging of the viral RNP but may also stimulate M oligomerization, as the viral RNP complex acts as a scaffold to concentrate M, helping it reach the threshold for particle formation. This critical event likely occurs at the ERGIC, where N, S, and M proteins have been experimentally demonstrated to accumulate in foci with the viral gRNA and ERGIC53, an ERGIC marker [47]. Indeed, budding of newly formed SARS-CoV-2 particles into single-membrane vesicles (SMVs) has been captured by electron microscopy [46], and these SMVs may be derived from the ERGIC, since ERGIC53 was detected by mass spectrometry in SARS-CoV-2 particles that were produced from Calu-3 cells [59].

### 5.2. SARS-CoV-2 Budding

Once assembled, SARS-CoV-2 must solve the universal problem faced by other enveloped viruses: closing the lipid membrane and completing budding. Some viruses, such as HIV-1, hijack the cellular endosomal sorting complexes required for transport (ESCRT) machinery to achieve budding [100,101,102,103,104,105,106]. The ESCRT machinery is involved in many cellular processes that require membrane scission away from the cytoplasm, such as endosomal sorting, cytokinesis, plasma membrane wound repair, nuclear membrane maintenance, and the repair of endo-lysosomal membranes [107,108,109,110]. HIV-1 and other viruses use short peptide motifs called late domains to interact with components of the ESCRT complexes and eventually recruit ESCRT-III and Vps4 to accomplish viral membrane scission and budding [101,102,103]. SARS-CoV-2, on the other hand, does not seem to recruit the ESCRT machinery for budding, because the dominant negative mutant of Vps4 which blocks HIV-1 budding does not compromise the production of SARS-CoV-2 VLPs [111].

ESCRT-independent budding, though less understood and more diverse, can either recruit other cellular factors or depend solely on viral proteins, and it has been reported in several other instances. A notable example is the mechanism of influenza A virus (IAV) ESCRT-independent budding, where membrane scission during budding at the plasma membrane is mediated by the viroporin M2 [79]. M2 insertion parallel to the lipid membrane via its amphipathic helix induces membrane curvature, leading to scission of the bud neck [78,112,113]. Interestingly, the use of amphipathic helices in an ESCRT-independent manner to induce membrane deformation is also employed by cellular proteins [114], including bar domain proteins and the Epsin protein family [115,116].

Although the mechanism by which SARS-CoV-2 and other coronaviruses bud remains elusive, several studies have suggested that the E protein may play a key role in inducing membrane curvature through an amphipathic helix in its highly conserved post-transmembrane region [111,117]. For instance, the mouse hepatitis virus (MHV) E protein was found to induce membrane curvature and be released in lipid vesicles when expressed in the absence of other structural proteins in mammalian cells, suggesting that E alone can drive the production of virus particles [117,118]. Similarly, in SARS-CoV-2, the E protein has remained the least mutated structural protein over the course of the COVID-19 pandemic, and it is indispensable for the production of infectious virus particles [48,49,111,119,120,121,122]. It is also reported that the SARS-CoV-2 E protein induces asymmetry between the two leaflets of the lipid bilayer, which increases membrane curvature and favors the formation of vesicles [71]. Nevertheless, research on the exact role of E during SARS-CoV-2 assembly and budding is still underway.

### 5.3. Egress and the Great Escape

After their assembly within intracellular membrane compartments, SARS-CoV-2 particles must exit producer cells to infect new target cells. Similar to other betacoronaviruses such as MHV, egress of SARS-CoV-2 does not depend on the conventional secretory pathway from the trans-Golgi to the plasma membrane, but rather appears to follow a lysosome-dependent pathway, as demonstrated by the co-localization of viral particles with lysosome marker LAMP1 [45]. Indeed, inhibiting the lysosomal GTPase Rab7 or Arl8b, key factors in lysosome-mediated secretion, impairs the release of both MHV and SARS-CoV-2 particles [45]. It is thus believed that SARS-CoV-2 particles in the ERGIC eventually reach lysosomes as they exit cells. In support of this egress strategy, SARS-CoV-2 uses its ORF3a and E proteins to deacidify lysosomes and inactivate lysosomal proteases [45,73,123].

While the mechanism by which SARS-CoV-2 particles reach the lysosomes after their formation at the ERGIC remains unclear, tracking the post-assembly modifications of these viral particles could provide valuable insights into this process. One such modification is protein glycosylation on the S and other viral proteins. Protein glycosylation begins at the ER with the conjugation of high-mannose glycans, and these are subsequently trimmed at the cis-Golgi, followed by the acquisition of complex glycans upon transiting through the medial- and trans-Golgi [124]. Importantly, when SARS-CoV-2 particles form in the ERGIC, spike and other viral structural proteins only bear high-mannose glycans, whereas upon successful release from cells, virion-associated S protein has been shown to bear mainly complex-type glycans [82,125]. Without this modification in the Golgi apparatus, the abundant high-mannose glycans on the S protein would be detected by immune sensors such as mannose-binding lectin (MBL), which would then activate the complement pathway to trigger inflammation [126].

One challenge to the acquisition of complex glycans by SARS-CoV-2 spike is the fragmentation or dispersal of the Golgi apparatus observed in infected cells [47]. While the functionality of these Golgi fragments has not been directly examined in SARS-CoV-2-infected cells, a similar phenomenon has been reported for other viruses such as poliovirus, where Golgi dispersion upon infection did not block protein secretion [127]. Likewise, dispersing the Golgi with the microtubule inhibitor nocodazole does not inhibit protein secretion either [128,129]. SARS-CoV-2 may therefore exploit Golgi fragmentation as a strategy to better access Golgi-resident enzymes to acquire complex glycans, since viral particles averaging 100 nm in size may be too large to efficiently traverse through the lumen of the Golgi cisternae that are 20–70 nm in size [130]. In support of this possibility, one study demonstrated that SARS-CoV-2 virions are enriched in dispersed Golgi fragments, and that this fragmentation may occur through SARS-CoV-2-mediated downregulation of GRASP55 [131].

## 6. Regulation of SARS-CoV-2 Assembly

SARS-CoV-2, like other viruses, relies on viral and cellular factors to regulate each stage of its replication cycle, including assembly. The virus has thus evolved numerous mechanisms, like host shutoff to hijack cellular machinery, post-translational modifications (PTMs) of viral proteins which modulate their functions, and interactions with host factors to facilitate efficient virion assembly and release.

Host shutoff suppresses the translation of cellular mRNAs to maximize the availability of cellular resources for the synthesis of viral proteins, including structural proteins [132,133]. A key SARS-CoV-2 protein in this process is NSP1, which binds to the 40S ribosomal subunit to block the access of cellular mRNAs, allowing only SARS-CoV-2 RNA to bypass this blockade and efficiently engage ribosomes for viral protein synthesis [134,135,136]. After SARS-CoV-2 release into the cytosol, the viral NSPs 2–16 also facilitate viral RNA replication by significantly remodeling host ER membranes into DMVs [39,55].

The functions of SARS-CoV-2 structural proteins in viral assembly are also subject to regulation by PTMs. For example, appropriate glycosylation of spike, which was previously described, is not only important for immune evasion but also for proper folding and intracellular trafficking for assembly [137]. Both S and E proteins also contain a cysteine-rich region adjacent to their TM domain, which undergoes palmitoylation [138,139,140]. The conjugated palmitoleic acid is inserted into the lipid membrane, directing the membrane topology of the S and E proteins, and concentrating S and E to the same membrane domains [137,138,139]. Furthermore, the N protein has been shown to acquire multiple types of PTMs, including phosphorylation and arginine methylation [141]. Between the independently folded N-terminal RNA-binding domain and the C-terminal dimerization domain of the N protein is a disordered Ser/Arg-rich region that is phosphorylated, and this has been shown to be important for viral replication and immune evasion as a result of regulating N-RNA condensates and the self-associating function of a leucine-rich helix [142,143]. Also in the N, two arginine residues R95 and R177 in the context of the RGG motifs undergo methylation by protein arginine methyltransferases, which regulates N binding to viral RNA and its ability to disrupt stress granules [144]. Additionally, the M protein has been shown to undergo ubiquitination at K15 by the ER ubiquitin ligase RNF5, which was found to promote stronger M-E interactions and efficient virus assembly and release [145].

The assembly function of SARS-CoV-2 structural proteins may also be mediated by a rich list of cellular proteins with which they interact, as revealed by proteomic studies [11,59,146,147]. It is conceivable that some of these host factors are able to modulate SARS-CoV-2 assembly through regulating the functions of these viral structural proteins. While more details are yet to be unveiled by future studies, some examples have been reported. One example is the stress granule proteins G3BP1/2 that interact with the N protein via their “ITFG” motif and get incorporated into SARS-CoV-2 particles, likely favoring the recruitment of N-RNA complexes to virus assembly sites containing the S protein (Figure 3) [59]. Efforts are underway to develop drugs that work by binding to G3BP1/2 more effectively than the SARS-CoV-2 N protein, aiming to disrupt the assembly of RNPs. However, these are early-stage genomic and in silico studies that require further validation through detailed characterization and clinical trials [148].

Viruses rely on both viral and host factors to facilitate their assembly. Paradoxically though, some host factors can also serve as restrictors or inhibitors, demonstrating the tug-of-war between viral strategies and host defense mechanisms. Several cellular proteins that have been reported to restrict the assembly of other RNA viruses were also shown to inhibit the production of infectious SARS-CoV-2 particles (Figure 3). For instance, BST-2 and SERINC5 inhibit SARS-CoV-2 release and viral infectivity, respectively [149,150]. In addition, the cellular RNA helicase MOV10 was shown to interact with the N protein of SARS-CoV-2 and other human coronaviruses, exhibiting antiviral activity through the sequestration of viral RNA in cytoplasmic granules [151]. Furthermore, cellular nucleic acid-binding protein (CNBP) competes with the viral N protein in binding to viral RNA and prevents the viral RNA and N protein from forming liquid—liquid phase separation condensates [152]. Genome-wide functional screens have provided a comprehensive view of cellular proteins that either promote or suppress SARS-CoV-2 replication, although data remain limited due to assay design challenges in measuring SARS-CoV-2 assembly [11,149,153,154]. Nevertheless, a screen of interferon-stimulated genes (ISGs) does reveal a short list of ISG products, particularly those involved in endoplasmic reticulum-associated degradation (ERAD) and vesicle trafficking pathways, that exhibit inhibitory effects on the late assembly stage of SARS-CoV-2 replication [149]. Further studies are warranted to validate these findings.

In addition to SARS-CoV-2 structural proteins, viral accessory and non-structural proteins also play significant roles in promoting viral assembly by modulating subcellular environments to favor virus production or by countering host cell restriction. A notable example of the latter is the deacidification of lysosomes by accessory factor ORF3a to warrant successful egress of SARS-CoV-2 particles [45,123]. As a second example, SARS-CoV-2 accessory protein ORF7a counters the cellular restriction factor BST-2, which would otherwise inhibit the release of virus particles [149,155]. ORF7a has also been shown to block the incorporation of cellular protein SERINC5 into SARS-CoV-2 particles, a restriction factor that inhibits membrane fusion during virus entry [150] (Figure 3). Interestingly, a recent mass spectrometry analysis of SARS-CoV-2 virions indicates the presence of ORF7a in the particles [59].

After evading the intracellular restriction factors, nascent SARS-CoV-2 particles face extracellular antiviral molecules, particularly antibodies which target the highly antigenic S protein and can neutralize virus particles. These immune pressures select for escape mutations in the S protein and other viral structural proteins, and play a key role in driving the continuous emergence of SARS-CoV-2 variants. In turn, these escape mutations in viral structural proteins may affect the assembly and infectivity of viral progenies, consequently impacting viral pathogenicity.

## 7. Conclusions

Producing infectious SARS-CoV-2 particles is a complex task that involves more than just the four viral structural proteins (M, E, N, and S) and the viral genomic RNA. Instead, it requires the coordination of their congregation at the right subcellar compartments, precise timing, and proper amounts of each component. A myriad of cellular factors, such as proteins and lipids, are assimilated by the virus to assist in the assembly process, often through their interactions with essential viral components. Simultaneously, SARS-CoV-2 must successfully counteract cellular factors that target and inhibit the production of infectious viral particles. Elucidating the molecular details of SARS-CoV-2 assembly and identifying the involved cellular factors requires an experimental system that is able to accurately recapitulate the assembly process taking place during viral replication. More is expected to be learned from the SARS-CoV-2 VLP assay using the luciferase mRNA reporter as the functional readout of infectivity, such as about the interactions of structural proteins essential for assembly, protein motifs orchestrating the trafficking of structural proteins to the assembly site, and the mechanism of viral RNA packaging. This assay can be adapted for screening both pro- and anti-assembly cellular factors and characterizing the functions of viral structural proteins in greater detail. Given the simplification of the VLP system which bypasses the earlier steps of viral replication, results from VLP assays will need to be validated in experiments with live SARS-CoV-2. Ultimately, exciting discoveries are expected regarding this important viral process, which will not only enrich our knowledge of SARS-CoV-2 replication, but also offer a promising strategy for antiviral drug development, potentially inhibiting viral replication and reducing infection rates. Moreover, this approach could lead to the development of broad-spectrum antivirals capable of combating not only SARS-CoV-2 but other related viruses as well.

## Data Availability

Data sharing is not applicable.
